# Ficolin-2 amplifies inflammation in macrophage-smooth muscle cell cross-talk and increases monocyte transmigration by mechanisms involving IL-1β and IL-6

**DOI:** 10.1038/s41598-023-46770-0

**Published:** 2023-11-08

**Authors:** Răzvan Daniel Macarie, Monica Mădălina Tucureanu, Letiția Ciortan, Ana-Maria Gan, Elena Butoi, Ileana Mânduțeanu

**Affiliations:** Biopathology and Therapy of Inflammation Department, Institute of Cellular Biology and Pathology “Nicolae Simionescu”, Bucharest, Romania

**Keywords:** Cardiovascular diseases, Mechanisms of disease

## Abstract

Ficolin-2, recently identified in atherosclerotic plaques, has been correlated with future acute cardiovascular events, but its role remains unknown. We hypothesize that it could influence plaque vulnerability by interfering in the cross-talk between macrophages (MØ) and smooth muscle cells (SMC). To examine its role and mechanism of action, we exposed an in-vitro co-culture system of SMC and MØ to ficolin-2 (10 µg/mL) and then performed cytokine array, protease array, ELISA, qPCR, Western Blot, and monocyte transmigration assay. Carotid plaque samples from atherosclerotic patients with high plasma levels of ficolin-2 were analyzed by immunofluorescence. We show that ficolin-2: (i) promotes a pro-inflammatory phenotype in SMC following interaction with MØ by elevating the gene expression of MCP-1, upregulating gene and protein expression of IL-6 and TLR4, and by activating ERK/MAPK and NF-KB signaling pathways; (ii) increased IL-1β, IL-6, and MIP-1β in MØ beyond the level induced by cellular interaction with SMC; (iii) elevated the secretion of IL-1β, IL-6, and CCL4 in the conditioned medium; (iv) enhanced monocyte transmigration and (v) in atherosclerotic plaques from patients with high plasma levels of ficolin-2, we observed co-localization of ficolin-2 with SMC marker αSMA and the cytokines IL-1β and IL-6. These findings shed light on previously unknown mechanisms underlying ficolin-2–dependent pathological inflammation in atherosclerotic plaques.

## Introduction

Atherosclerosis is a chronic, lipid-driven vascular inflammatory disease, characterized by the formation of an atherosclerotic plaque (atheroma or fibroinflammatory lipid plaque) in the vessel wall of medium- or large-sized elastic or muscular arteries, thereby impairing arterial function.

The development of atherosclerotic plaques is a gradual and continuing process which involves a complex cross-talk between resident and migrated cells, as well as inflammatory mediators present in the arterial wall, ultimately resulting in plaque rupture and atherothrombosis^1^. Smooth muscle cells (SMC) often locate adjacent to macrophage (MØ) clusters and the dynamic interplay between MØ, SMC and their surrounding microenvironment plays a significant role in atherosclerotic plaque evolution and stability^2^. Recent advances in the field of bioinformatics, particularly in single-cell technologies provided new data on the transcriptome of individual cells and confirmed the cellular cross-talk within atherosclerotic plaques^3^. We have previously shown that the interaction between macrophages and smooth muscle cells considerably amplifies macrophage cytokine expression with consequences on leukocyte recruitment^4^. Accordingly, in a recent work, it was found that inflammatory mediators significantly modulate SMC and macrophage cross-talk and affect plaque stability^2^. In addition, we demonstrated that the cross-talk between macrophages and smooth muscle cells disrupted the synthesis of collagen and metalloprotease and promoted angiogenesis. These changes could impact the progression of atheroma towards a vulnerable plaque^5^.

Vulnerable plaques are responsible for major cardiovascular events, including ischemic stroke. Unfortunately, current methods to identify high-risk patients are limited to invasive or non-invasive imaging techniques, such as intravascular ultrasound or computed tomographic angiography, as there are no known biomarkers for detecting rupture-prone vulnerable plaques^6^. However, evidence suggests that the complement system, due to its pro-inflammatory reactions, may contribute to the erosion of atherosclerotic plaques. Furthermore, the levels of complement proteins in the blood might serve as markers for cardiovascular and stroke risk^7,8^.

The initiation of the complement lectin pathway (LP) involves recognition molecules such as mannose-binding lectins (MBL), ficolins, and collectins, which form complexes with the proteolytic enzymes of the mannan-binding lectin serine protease (MASP). These proteins have been identified as significant contributors to the progression of atherosclerotic plaques^9^. Furthermore, correlations have been established between circulating complement proteins, plaque morphology, and an elevated risk for ischemic stroke^7^. Notable, ficolin-2 was found to be present in human carotid atherosclerotic plaque, detected in cholesterol-enriched plaque regions in association with macrophages, and correlated with the vulnerable plaque^7,10^. Patient screening data indicated a positive correlation between ficolin-2 and elevated levels of circulating inflammatory markers, as well as intraplaque immune cell recruitment. Moreover, ficolin-2 was demonstrated to predict MACE (Major Adverse Cardiac Events) in atherosclerotic patients^10^, but it is not clear if ficolin-2 is merely present in the plaque due to an immune response or if it actively contributes to plaque progression by affecting cells and regulating molecules essentially involved in plaque progression. Therefore, the present study was developed in order to find a possible role of ficolin-2 in this pathology, by uncovering the cellular and molecular mechanisms involved. Previous research had demonstrated that mouse ficolin-A (a molecule closely related to human ficolin-2) induces pro-inflammatory cytokines IL-6, IL-1β and TNFα and activates MAPK/NF-kB signaling pathways in mouse monocytic cell line Raw264.7 and THP-1-differentiated macrophages^11^.

In the present study, we hypothesize that ficolin-2 might interfere with the communication between macrophages and smooth muscle cells.

## Materials and methods

### Materials

Proteome Profiler Human Cytokine Array Kit, Proteome Profiler Human Protease Array Kit, Human IL-1β and Human IL-6 DuoSet ELISA kits, recombinant human ficolin-2 protein, mouse monoclonal anti-human IL-1β antibody, goat polyclonal anti-human MIP-1β antibody, mouse monoclonal anti-human ERK1/2 antibody, mouse monoclonal anti-human Toll-like receptor 4 (TLR4) antibody and rabbit polyclonal anti-human p38 antibody were purchased from R&D Systems. Monoclonal rabbit anti-human IL-6 antibody, monoclonal mouse anti-human phospho-ERK1/2 antibody, monoclonal rabbit anti-human phospho-p65 antibody, monoclonal rabbit anti-human phospho-p38 antibody, polyclonal rabbit anti-human JNK antibody, monoclonal mouse anti-human phospho-JNK antibody and monoclonal mouse anti-human β-actin antibody were purchased from Cell Signaling Technology. Mouse monoclonal anti-human ficolin-2 antibody coupled with FITC was purchased from Novus Biologicals. Mouse monoclonal anti-human ficolin-2, polyclonal rabbit anti-human p65 antibody, biotin-coupled and AlexaFluor594-coupled secondary antibodies, Human Vascular Smooth Muscle Cell Basal Medium, Smooth Muscle Growth Supplement and all the reagents used for molecular biology were purchased from Thermo Fisher Scientific. Monoclonal mouse anti-human α-actin antibody was purchased from Santa Cruz Biotechnology, permeable supports for 6-well plates with 0.4 µm PET membrane were from BD Falcon, primers were synthesized by Eurogentec and all other reagents and chemicals were from Sigma Aldrich, Merck and Thermo Fisher Scientific.

### Cells

Human aortic smooth muscle cells (HAoSMC) (from Thermo Fisher Scientific), positive for smooth muscle cell-specific alpha-actin (αSMA) expression, were grown in smooth muscle cell growth medium containing 10% fetal bovine serum, 100 U/ml penicillin and 100 µg/ml streptomycin in a humidified incubator at 37°C with 5% CO_2_. THP-1 human monocytic cells (from ECACC) derived from an acute monocytic leukaemia patient were cultivated at 37°C in RPMI medium containing 10% heat-inactivated FBS, 100 U/mL penicillin and 100 μg/mL streptomycin in a humidified incubator with 5% CO_2_. THP-1 cells, a cell line extensively studied as a model for human macrophages^12^, were differentiated into macrophages (MØ) with 100 nM phorbol 12-myristate 13-acetate (PMA) for 72 h before experiments.

### Experimental design

To establish the SMC-MØ co-culture, SMC were cultured on the lower chamber of a transwell system until confluence and THP-1 monocytes were differentiated to MØ directly on the transwell filter membrane (0.4 µm pore size) using 100 nM PMA for 72 h. After MØ were differentiated and SMC reached confluence, the inserts were placed in the wells, and the co-culture was maintained in RPMI serum-free medium. To examine the role of ficolin-2, recombinant human protein at a concentration of 10 µg/ml was added to the co-culture (both lower and upper chamber) and incubated for 1 h, 6 h or 24 h. The optimal concentration of ficolin-2 was pre-determined in previous studies^11^. The conditioned media and the cells were isolated and prepared for further analysis.

### Human protein array and image analysis

To assess cytokines and proteases expression profiles in the interaction between SMC and MØ in the presence or absence of ficolin-2, we used Proteome Profiler Human Cytokine and Protease Array kits (R&D Systems), capable of detecting the relative expression levels of 36 cytokines and 35 proteases, respectively. Briefly, the nitrocellulose membranes containing capture antibodies were incubated overnight at 4°C with a mixture of the sample consisting of conditioned media (from SMC, MØ, SMC-MØ co-culture in the presence or absence of ficolin-2 recombinant protein) and biotinylated detection antibody cocktail. After several washing steps, the membranes were incubated with streptavidin conjugated to HRP (horseradish peroxidase) and then exposed to HRP substrate. Images were captured with ImageQuant LAS 4000 (GE Healthcare) and the optical integrated density was calculated using ImageJ software, each spot being normalized to the average intensity of reference spots. The experiment was performed in duplicate.

### RNA isolation and real-time PCR

Total cellular RNA was isolated from interacted or non-interacted MØ and SMC in the presence or absence of ficolin-2 recombinant protein using PureLink RNA Mini kit (ThermoFisher Scientific). First-strand cDNA synthesis was performed employing 1 µg total RNA and MMLV reverse transcriptase according to the manufacturer's protocol (ThermoFisher Scientific). Gene expression quantification was performed by cDNA amplification using a LightCycler 480 Real-Time PCR System (Roche), SYBR Select Master Mix (ThermoFisher Scientific), and specific primer pairs, listed in Supplemental material Table S1. The expression of analyzed genes was normalized to β2-microglobulin mRNA level. The relative quantification was performed using the comparative cT method.

### Enzyme-linked immunosorbent assay (ELISA)

The concentration of IL-1β and IL-6 in the conditioned media of SMC-MØ in the presence/absence of ficolin-2 was measured by ELISA, using DuoSet kits (R&D Systems) according to the manufacturer’s protocol. Briefly, the capture antibody was coated on a 96-well microplate overnight. After three washes, the plate was blocked with 1% BSA (bovine serum albumin). Samples and standards were added to the plate and incubated for 2 h at room temperature, followed by a 2 h incubation with the detection antibody. To measure the concentration of inflammatory mediators, the samples were sequentially incubated with Streptavidin-HRP for 20 min, substrate solution (with H_2_O_2_ and tetramethylbenzidine), and stop solution (2N H_2_SO_4_). The optical density at 450 nm was determined using an Infinite 200 Pro Tecan microplate reader, with wavelength correction at 540 nm.

### Protein expression analysis by Western Blot

Protein expression of IL-6, IL-1β, MIP-1β, TLR4, phospho-p65/p65-NF-kB, phospho-ERK/ERK, phospho-p38/p38, phospho-JNK/JNK and β-actin was assessed in interacted or non-interacted SMC and MØ, in the presence or absence of ficolin-2. Cells were washed with PBS (phosphate buffer saline) before adding RIPA lysis buffer (25 mM Tris–HCl pH 7.6, 150 mM NaCl, 1% NP-40, 1% sodium deoxycholate, 0.1% SDS, 5 mM EDTA, protease and phosphatase inhibitors) and gently rocked at 4°C. Lysed cells were centrifuged at 15.000 × *g* for 5 min and the supernatant was transferred into clean tubes and loaded using Laemmli electrophoresis buffer (5×) in 10–12% SDS-PAGE gels. Gels were blotted on nitrocellulose paper and specific primary antibodies and secondary HRP-conjugated antibodies were used. The chemiluminescent substrate SuperSignal West Dura (from ThermoFisher Scientific) was visualized using the ImageQuant LAS 4000 imaging system (GE Healthcare) and quantified by densitometry using the ImageJ software.

### Immunofluorescence

To investigate whether ficolin-2 interacts with SMC or MØ, both cell types were treated with 10 µg/ml ficolin-2 recombinant protein for 24 h, and the binding of ficolin-2 was assessed by immunofluorescence. Cultured SMC or differentiated MØ were fixed with 4% paraformaldehyde, permeabilized with PBS + 0.5% Triton X100 for 20 min and blocked with 3% bovine serum albumin (BSA), 1% fish gelatin and 0.2% Tween 20. Cells were incubated overnight with primary anti-human ficolin-2 antibody, followed by incubation with chicken anti-mouse AlexaFluor594 conjugated secondary antibody (ThermoFisher Scientific). Nuclei were stained with 4′,6-diamidino-2-phenylindole (DAPI), F-actin was labeled with fluorescein phalloidin and cell membrane was stained using AlexaFluor488 wheat germ agglutinin (WGA). Images were acquired using the fluorescence microscope Olympus IX8 equipped with an XM10 camera and the confocal microscope Leica TCS-SP5and processed using ImageJ software.

For intraplaque co-immunolabeling of IL-1β, IL-6 and αSMA with ficolin-2, we used frozen sections from three carotid plaque specimens of patients with severe carotid stenosis from a human study described in^10^. The sections were prepared according to the protocol described by Carbone et al.^10^. Frozen plaque sections were first fixed with 4% paraformaldehyde and blocked with 3% bovine serum albumin for 1 h. The samples were incubated overnight at 4°C with either anti-IL-1β, IL-6 or αSMA antibodies. After primary antibody incubation, the sections were washed and incubated with AlexaFluor594-conjugated secondary antibodies for 1 h, washed again and incubated with FITC-coupled ficolin-2 antibody. Nuclei were counterstained with DAPI. Stained sections were visualized under the fluorescence microscope, focusing on the necrotic core of the atherosclerotic plaque. Images were acquired and processed using CellSens Dimensions and ImageJ software.

### Determination of cell migration

Real-time migration of monocytes was monitored using CIM-plate-16 and the xCELLigence System RTCA DP Instrument (Roche). We used a 16-well modified Boyden chamber composed of an upper chamber (UC) and a lower chamber (LC) that snapped together to form a tight seal. The bottom of the UC consists of a microporous polyethylene terephthalate membrane that permits the translocation of cells from the upper to the bottom side. Cell migration was monitored by interdigitated gold microelectrode sensors that generate an impedance signal by contact with the migrated cells. Conditioned media from SMC-MØ co-culture in the presence or absence of ficolin-2 was added to the LC. We seeded 10 × 10^5^ monocytes in the UC of the CIM-plate-16 in RPMI medium without FCS. Cell migration was monitored for up to 6 h.

### Statistical analysis

All statistical analysis was performed with GraphPad Prism 7.0 software, with data points representing mean ± standard error (SE). Statistical significance is shown as p-values obtained with a one-sample t-test, two-tailed Student’s t-test or one-way ANOVA, as appropriate. A p-value of p < 0.05 was considered statistically significant.

## Results

### Ficolin-2 binds to macrophages and SMC in vitro and is associated with SMC in the plaques of atherosclerotic patients with high plasma levels of ficolin-2

**Figure 1:**
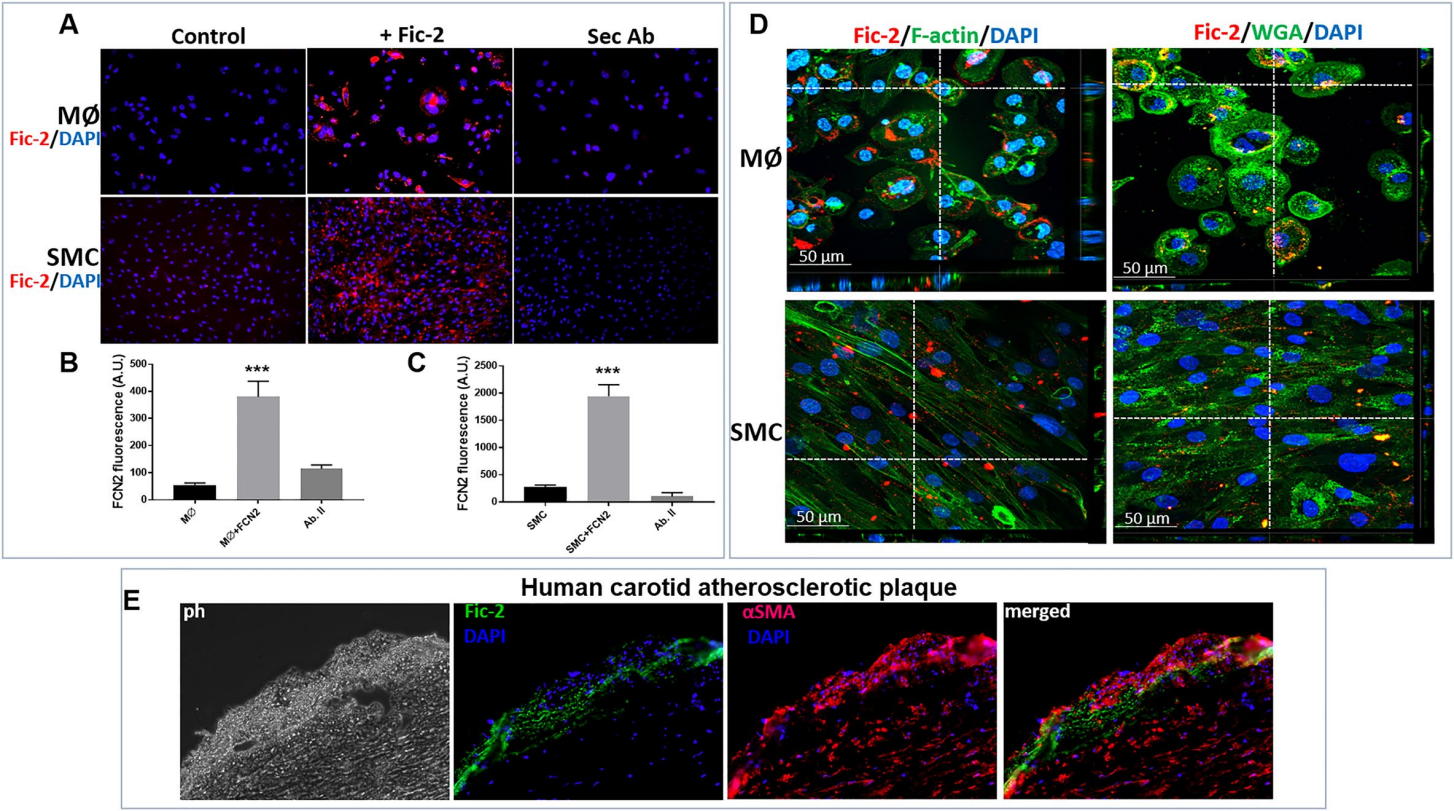
Ficolin-2 interacts with MØ and SMC. (**A**) Immunofluorescence images display the interaction of ficolin-2 (Fic-2) with MØ or SMC, using an anti-ficolin-2 antibody as the primary antibody, and a secondary antibody coupled with AlexaFluor594. DAPI was used for nuclear staining. (**B**) Quantification of ficolin-2 interacting with MØ treated with ficolin-2 for 24 h, compared with control MØ (untreated). (**C**) Quantification of ficolin-2 interacting with SMC treated for 24 h with ficolin-2, compared with control SMC. The data is represented as arbitrary units of fluorescence intensity. An anti-mouse AlexaFluor594 secondary antibody was used as a negative control. *** represents p < 0.001 as analyzed by unpaired Student’s t-test on treated vs. non-treated, n = 3. (**D**) Confocal microscopy shows the distribuition of ficolin-2 at the cellular level after treating MØ and SMCs with the recombinant protein for 24 h. Cells were counterstained with either phalloidin-FITC to label F-actin or AlexaFluor488-wheat germ agglutinin (WGA) to label cell membrane. Nuclei were stained using DAPI. (**E**) Immunofluorescence images showing the presence of ficolin-2 and SMC marker αSMA on sections obtained from the necrotic core region of the atherosclerotic plaque.

Previously it was demonstrated that in vitro, ficolin-2 binds to macrophages, dendritic cells and CD8^+^ T cells, but not to CD4^+^ T cells in mice^13^. We examined whether ficolin-2 directly interacts with both types of cells used in our experimental model, specifically MØ and SMC. Using a human anti-ficolin-2 antibody, we analyzed by immunofluorescence whether ficolin-2 interacts with SMC or MØ after a 24 h incubation period with the recombinant human protein at a concentration of 10 µg/ml. Our data demonstrate that ficolin-2 added in the culture media interacts with both macrophages (Fig. 1A and B) and smooth muscle cells (Fig. 1A and C).

Confocal microscopy imaging shows the distribution of ficolin-2 in the cytosol and on the cell membrane, both for macrophages and SMC (Fig. 1D).

Recent studies detected the presence of ficolin-2 within the plaque necrotic core of patients with atherosclerosis^10^, in areas rich in cholesterol crystals, and co-localized with CD11b, a surface marker of macrophages. To corroborate our in vitro observations of ficolin-2 binding to SMC, we investigated the presence of SMC and ficolin-2 within the plaque. The specimens of atherosclerotic plaques within the necrotic core area were positive for both αSMA and ficolin-2, with ficolin-2 localization in close proximity to SMC in the atherosclerotic plaque (Fig. 1E and Supplementary material Figure S1).

### Ficolin-2 effect on SMC

#### Ficolin-2 does not directly activate SMC

**Figure 2:**
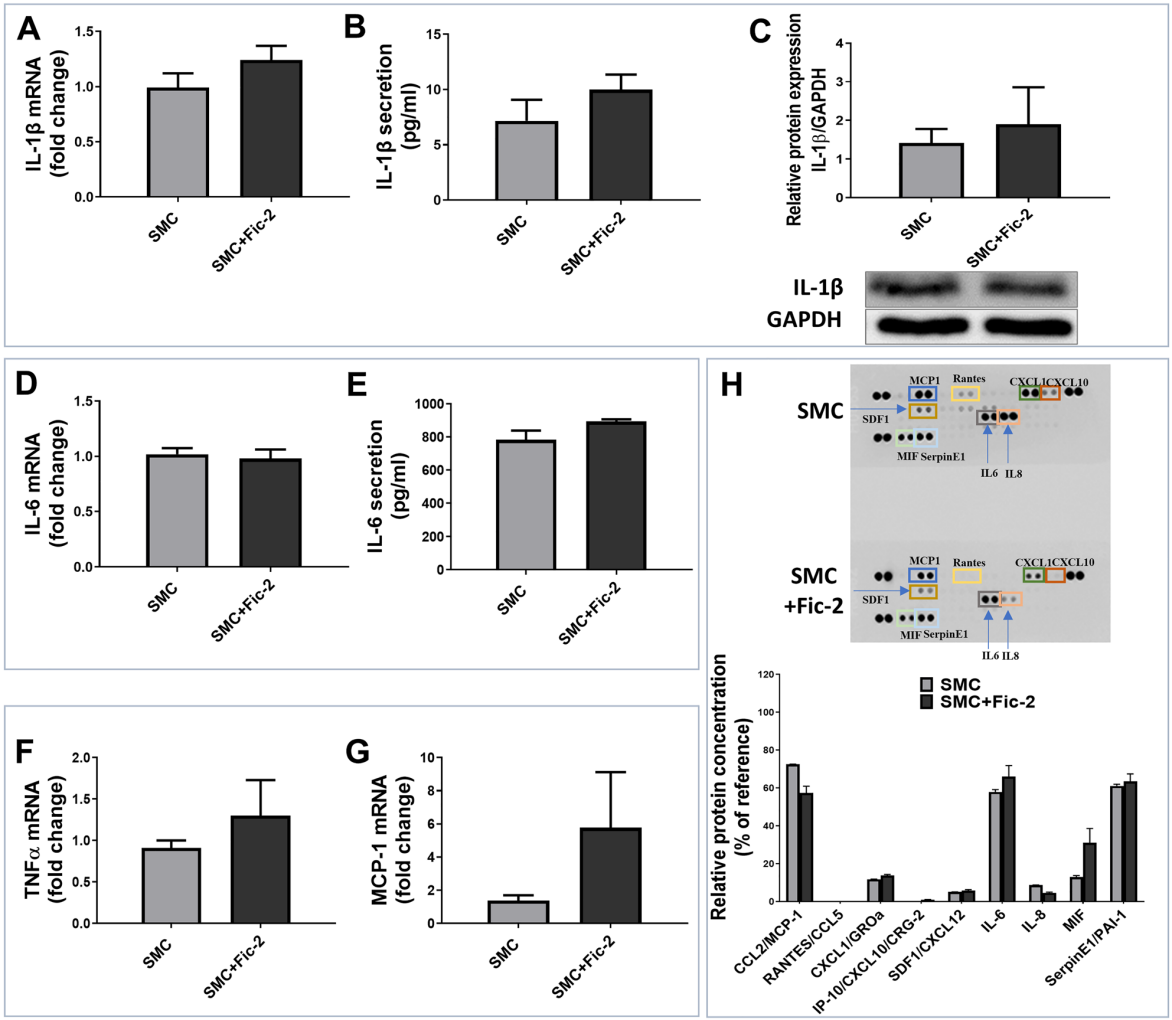
Ficolin-2 does not significantly alter the inflammatory profile of SMC after 24 h. Gene expression of inflammatory molecules IL-1β (**A**), IL-6 (**D**), TNFα (**F**) and MCP-1 (**G**) were analyzed in SMC exposed to ficolin-2 for 24 h; values are expressed as fold change over control SMC (n = 3). The secretion of IL-1β (**B**) and IL-6 (**E**) was assessed by ELISA and expressed as pg/ml of secreted protein (n = 3). (**C**) Western Blot analysis of IL-1β in ficolin-2 exposed SMC and control SMC is expressed as relative protein expression, normalized by GAPDH expression (n = 3). (**H**) Representative images from the Cytokine Proteome Profiler Array and dot-blot quantification of secreted proteins in the conditioned media.

Given that ficolin-2 demonstrated binding capabilities to SMC in vitro (Fig. 1C and D) and that prior studies have shown that ficolin-2 induces an inflammatory M1 phenotype in macrophages^13^, we decided to evaluate the impact of ficolin-2 on SMC inflammation. Therefore, we exposed SMC to ficolin-2 and evaluated gene expression, protein expression and secretion of several typical inflammatory mediators like IL-1β, IL-6, TNF-α and MCP-1. We found that exposure to ficolin-2 did not significantly upregulate any of these mediators at the gene or protein level, nor did it affect secretion in SMC (Fig. 2A–G). Moreover, the screening of a panel of 36 cytokines using a Human Cytokine Proteome Profiler Array indicated that the levels of analyzed cytokines remained unmodified in SMC-conditioned media after ficolin-2 exposure (Fig. 2H). Together, these data suggest no direct impact of ficolin-2 on SMC, despite ficolin-2 being detected on the SMC surface (Fig. 1 D). Thus, the absence of significant changes led us to explore the possibility that ficolin-2 might impact SMC through some indirect mechanisms.

#### Ficolin-2 activates SMC upon their cross-talk with macrophages

**Figure 3:**
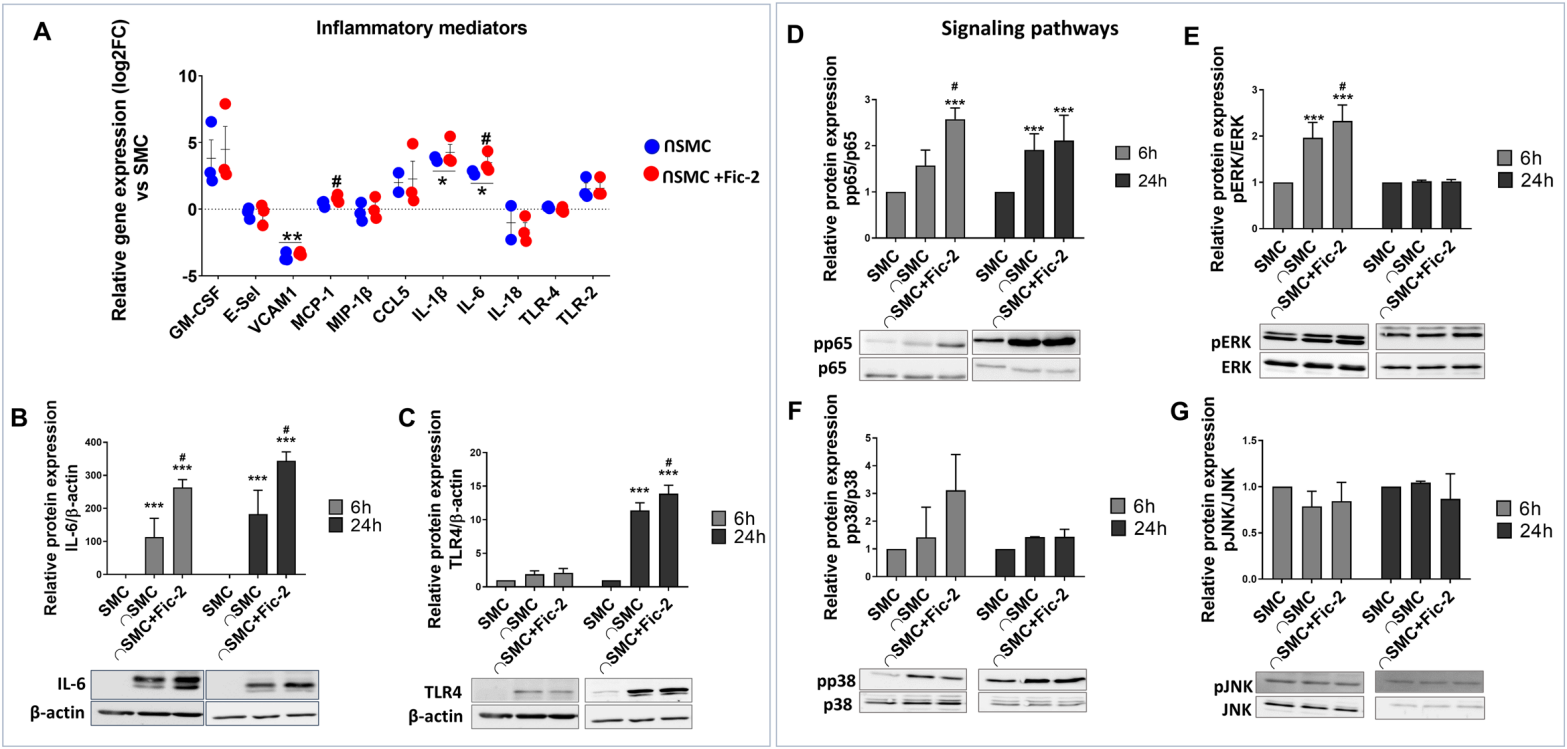
Ficolin-2 increases MCP-1 and IL-6 gene expression, IL-6 and TLR4 protein expression, and stimulates the activation of NF-kB and ERK/MAPK pathways in SMC-MØ co-culture. (**A**) Gene expression of inflammatory molecules reported as log2FC vs SMC (dotted line). (**B**) Western Blot analysis shows that ficolin-2 significantly increased IL-6 protein expression after 6 h and 24 h. (**C**) TLR4 receptor expression also sees a significant increase after 24 h of interaction. (**D**) Ficolin-2 activates the NF-kB transcription factor (p65 subunit) and (**E**) the ERK1/2 MAPK signaling pathway in SMC interacted with macrophages. (**F** & **G**). No activation was observed for the p38 and JNK MAPK signaling pathways. * p < 0.05, *** p < 0.001 vs SMC; # p < 0.05 vs interacted SMC (⋂SMC) using one-way ANOVA (n = 3).

Accumulating data indicate that SMC can modify their phenotype towards a remodeling, inflammatory, or osteogenic phenotype upon interaction with macrophages^14,15^. Moreover, there is evidence that ficolin-2 induces an M1 inflammatory phenotype in macrophages^13^. Given these findings, we evaluated the effects of ficolin-2 on SMC during their interaction with macrophages. Using qPCR analysis, we examined the expression of various inflammatory mediators in SMC, including GM-CSF, E-selectin, VCAM1, MCP-1, MIP-1β, CCL5, IL-1β, IL-6, IL-18, TLR-4, and TLR-2. Gene expression analysis of inflammatory markers revealed that following the 24-h interaction with macrophages (without ficolin-2), the gene expression of IL-1β and IL-6 was increased in SMC while VCAM-1 gene expression was decreased (Fig. 3A – blue dots). Exposure of interacted cells to ficolin-2 also led to an upregulation of MCP-1 and IL-6 gene expression over the levels induced only by cross-talk, whereas the gene expression of IL-1β remained unchanged (Fig. 3A – red dots).

Next, we assessed the protein expression of the molecules that exhibited increased gene expression levels using Western Blot analysis. Our results showed that ficolin-2 significantly increased the protein expression of IL-6 in SMC after both 6 h and 24 h of interaction with MØ (Fig. 3B) and the protein expression of TLR4 after 24 h interaction (Fig. 3C). However, exposure to ficolin-2 did not result in any noticeable modification of MCP-1 protein expression (data not shown).

We proceeded to examine the signaling pathways activated in SMC interacted with MØ upon exposure to ficolin-2. We could observe that ficolin-2 activated the NF-kB and ERK/MAPK signaling pathways after 6 h of interaction with macrophages. However, this activation seems to decline by the 24 h mark (Fig. 3D and E). In our experimental model, we did not observe any significant activation of p38 and JNK MAPK pathways in SMC after 6 or 24 h of interaction with macrophages (Fig. 3F and G). Additionally, we investigated the inflammasome signaling and found no evidence of NLRP3 or caspase-1 activation following stimulation of the SMC-MØ co-culture with ficolin-2 for either 6 h or 24 h (data not shown).

**Figure 4:**
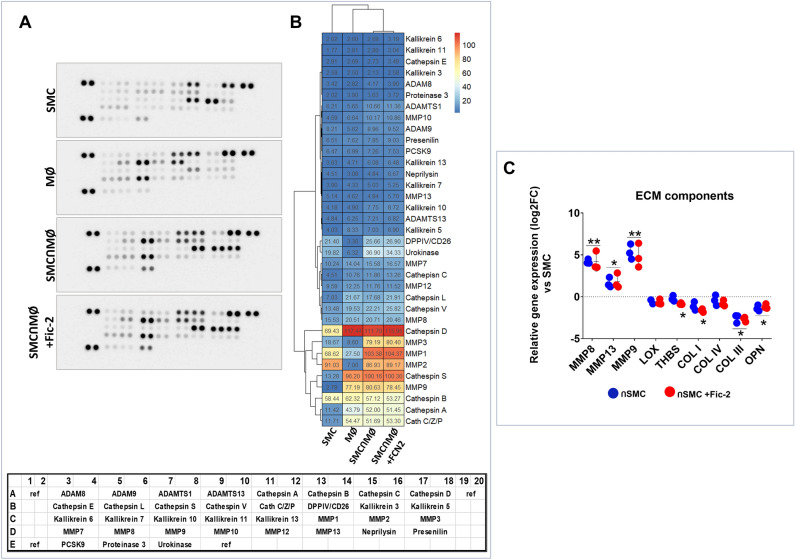
The SMC-MØ interaction results in altered protease levels in the conditioned media; ficolin-2 does not induce any further changes. (**A**) Proteases released into the conditioned medium of SMC-MØ in the presence of ficolin-2. On the left side, the Proteome Profiler membrane image is shown, with its specific spot intensity following incubation with conditioned media from SMC, MØ, SMC-MØ, and SMC-MØ + ficolin-2 (n = 2). (**B**) On the right side, a heatmap shows the relative intensity values for the specific spots, compared to the reference spots. (**C**) Relative gene expression (log2FC vs SMC) of MMPs and ECM components (dotted line). * p < 0.05 vs control SMC; ** p < 0.01 vs control SMC (n = 3).

To further investigate the effect of ficolin-2 on the proteases secreted in the co-culture-conditioned medium, we employed a Proteome Profiler kit. This allowed us to analyze 35 proteases released in the conditioned medium from the SMC-MØ co-culture, both in the presence or absence of ficolin-2. Non-interacted SMC and MØ were used as the negative control. In Fig. 4A and B it can be seen that a series of proteases, such as MMP3, MMP1, Cathepsin S, and Cathepsin A, were increased due to the SMC-MØ interaction. However, the presence of ficolin-2 did not significantly modify the levels of these or any other protease analyzed.

By qPCR, we assessed matrix proteases and ECM (extracellular matrix) components such as MMP8 (matrix metalloproteinase 8), MMP13, MMP9, LOX (Lysyl oxidase-1), THBS (thrombospondin 1), Col I (collagen I), Col IV (collagen IV), Col III (collagen III), and OPN (osteopontin). We observed that MMP8, MMP13, and MMP9 were increased and THBS, Col I, Col III, and osteopontin were decreased in SMC after their interaction with macrophages, while exposure to ficolin-2 did not modify this remodeling profile (Fig. 4C).

Concerning the phenotype markers of SMC and macrophages, we observed no significant modification in αSMA and CD68 gene expression in interacted SMC and ficolin-2 did not impact the expression of these molecules (Supplemental material – Figure S2).

### Ficolin-2 increases the protein expression of IL-1β, IL-6 and MIP-1β in MØ interacted with SMC and IL-1β, MIP-1α/β, IL-6 release in the conditioned media of the SMC-MØ co-culture

**Figure 5:**
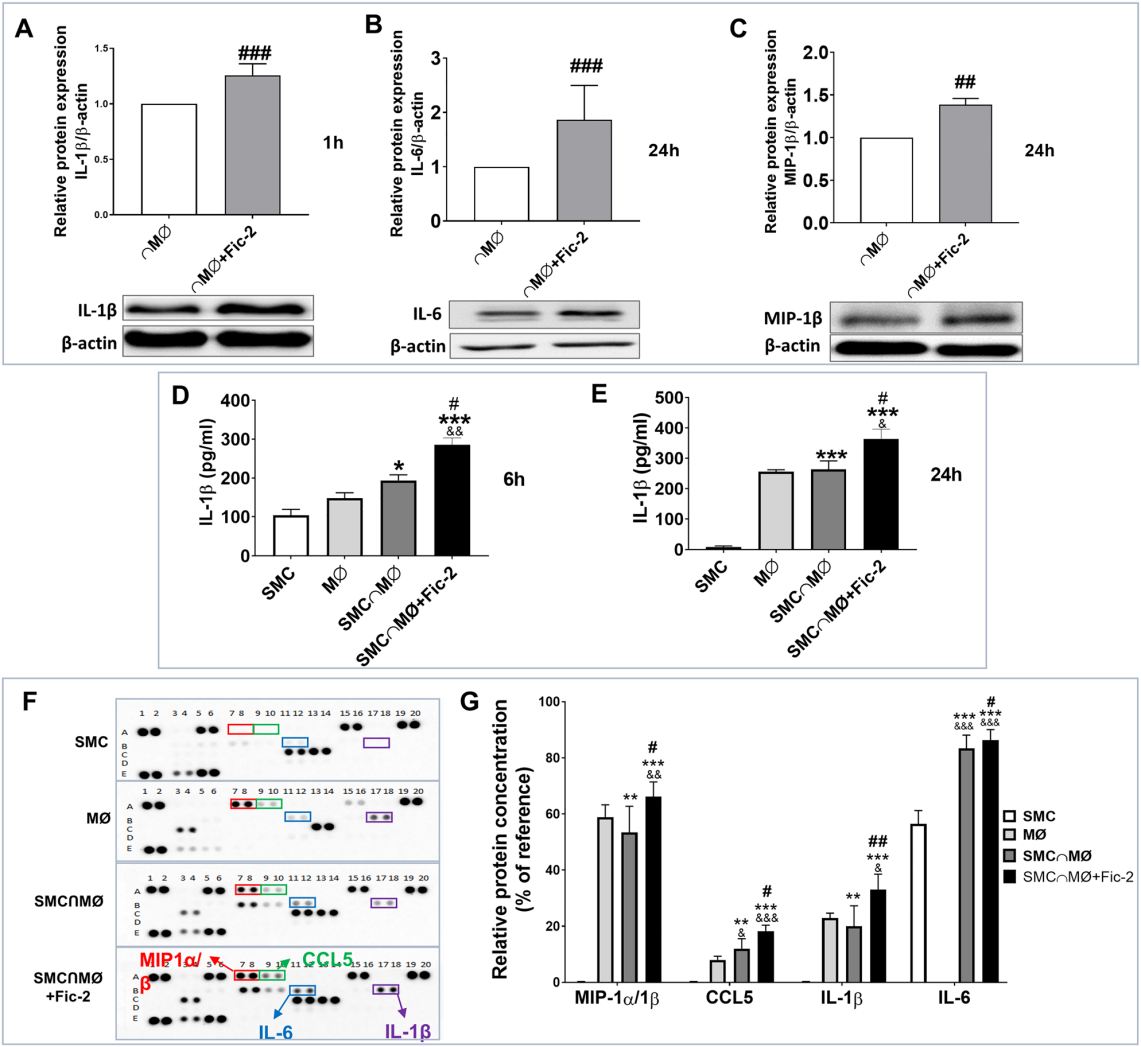
Ficolin-2 induces an increased expression of IL-1β, IL-6 and MIP-1β in MØ upon their cross-talk with SMC. (**A**) Western Blot analysis shows that in MØ, ficolin-2 significantly increased the expression of IL-1β protein expression after 1 h and (**B & C**) of IL-6 and MIP-1β after 24 h of interaction. (**D & E**) ELISA quantification of cytokine secretion in SMC⋂MØ and SMC⋂MØ + Fic-2 demonstrates a significant increase of IL-1β in the presence of ficolin-2 after 6 and 24 h. (**F**) Representative images of cytokine array in SMC, MØ, interacted SMC and MØ (SMC⋂MØ) and SMC-MØ interaction in the presence of ficolin-2 (SMC⋂MØ + Fic-2). Dots representing MIP-1α/β, CCL5, IL-1β and IL-6 are indicated in every blot. (**G**) Dot-blot quantification shows molecules significantly increased by ficolin-2. For images A, B and C, significance is denoted as ## p < 0.01 and ### p < 0.001 vs interacted MØ (⋂MØ) (n = 3). For images D and E, significance is represented as: * p < 0.05, ** p < 0.01, *** p < 0.001 vs SMC; & p < 0.05, && p < 0.01, &&& p < 0.001 vs MØ and # p < 0.05, ## p < 0.01 vs SMC⋂MØ (n = 3). Significance was determined using an unpaired Student’s t-test for graphs A, B and C and one-way ANOVA for graphs D, E and G.

Since it has been shown that ficolin-2 activates macrophages and enhances the production of inflammatory cytokines such as IL-1β, IL-6 and TNFα in THP-1-derived macrophages^11^, we further investigated the influence of ficolin-2 exposure on cytokine protein expression in macrophages within our SMC-MØ co-culture model. Using Western Blot assay we found an increase in IL-1β protein expression within macrophages after 1 h activation, and of IL-6 and MIP-1β after 24 h activation (Fig. 5A–C). Furthermore, by ELISA assay we detected a significant increase in IL-1β secretion in the conditioned media after both 6 and 24 h of cell interaction (Fig. 5D and E). This increase was significant when compared to the secretion levels seen during cell interaction without ficolin-2. Using a Human Cytokine Array kit for 36 cytokines (as described in “Human protein array and image analysis” section) we found that ficolin-2 significantly increased the secretion of IL-1β, MIP-1α/β, IL-6, and CCL5 in the conditioned medium of the SMC-MØ co-culture, compared with the untreated co-culture (Fig. 5F and G). However, the presence of CCL5 was not validated by subsequent gene and protein expression analysis (data not shown).

### Ficolin-2 intervention in SMC-MØ cross-talk determines an increase in monocyte chemotaxis

Given our observations that ficolin-2 enhances SMC and MO inflammation and the release of inflammatory cytokines, after SMC and MØ interaction, we further investigated ficolin-2 effects on monocyte chemotaxis.

We used the xCELLigence system to assess the ability of conditioned media (from SMC-MØ co-culture with or without ficolin-2) to induce monocyte transmigration. Monocytes were placed in the upper chamber of a Cell Invasion/Migration (CIM)-plate, while conditioned media from SMC-MØ interacted in the presence or absence of ficolin-2, was added to the lower chamber. The chemotactic activity was continuously monitored for 6 h using the xCELLigence software.

**Figure 6:**
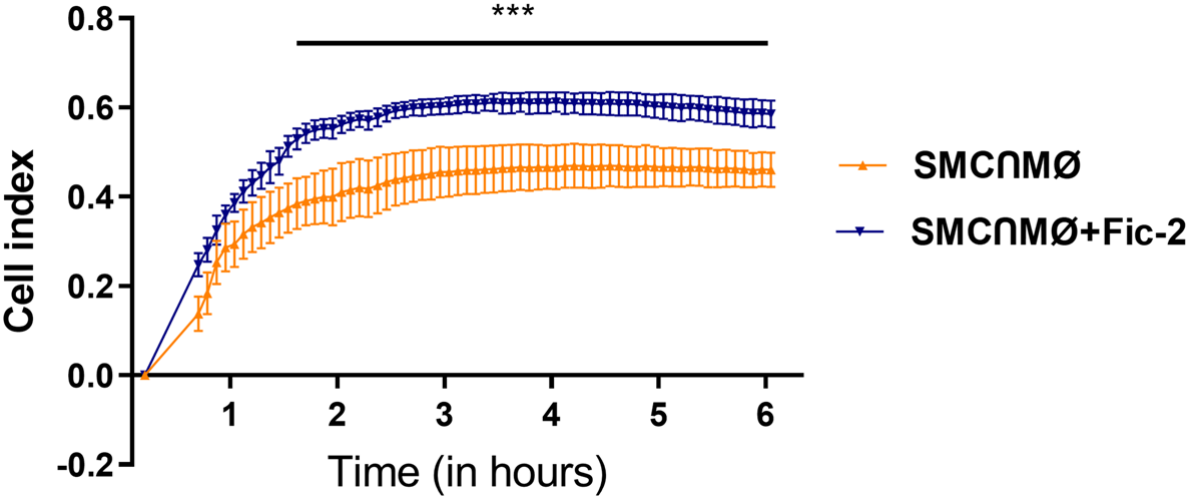
Ficolin-2 promotes monocyte chemotaxis. Evaluation of the chemotactic activity of monocytes toward conditioned media from SMC-MØ co-culture in the presence or absence of ficolin-2 was evaluated using the xCELLigence RTCA DP system. The cell index, which is proportional to the number of transmigrated monocytes, was measured as an electrical impedance every 5 min throughout 6 h. The data shown represent the mean fold change to control ± SE (n = 4) and are from two independent experiments with two replicates per experiment. ***p < 0.001.

As depicted in Fig. 6, the presence of ficolin-2 in the conditioned media of the SMC-MØ co-culture led to a larger number of migrating monocytes, compared to those migrating towards media from the SMC-MØ co-culture alone. This suggests that ficolin-2 can play a significant role in enhancing monocyte chemotaxis.

### Ficolin-2 is associated with IL-1β and IL-6 in the atherosclerotic plaque

**Figure 7:**
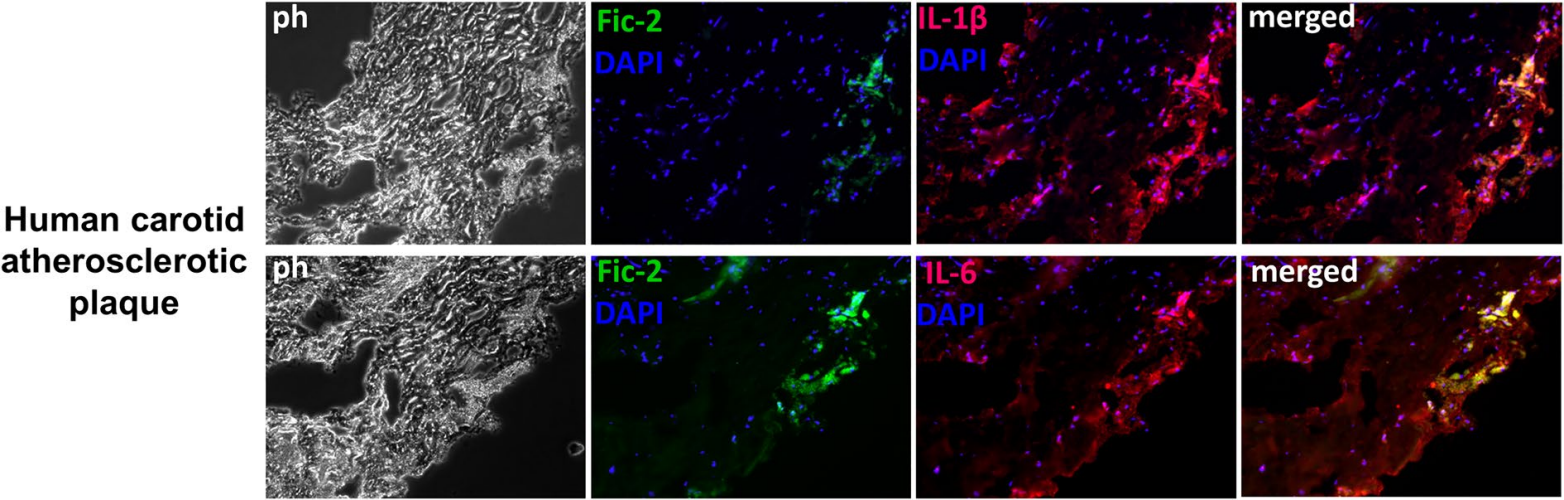
Ficolin-2 colocalized with IL-1β and IL-6 in the atherosclerotic plaque. Immunofluorescence staining and colocalization of ficolin-2 and IL-1β (upper panel) and ficolin-2 and IL-6 (lower panel) in specimens of human carotid atherosclerotic plaque from patients with high plasma levels of ficolin-2. Ficolin-2-FITC-coupled antibody (green), IL-1β and IL-6 primary antibodies coupled with an AlexaFluor594 secondary antibody (red) and DAPI for nuclear staining (blue) were used.

Since the presence of IL-1β and IL-6 cytokines in atherosclerotic plaque and their role in the amplification of inflammatory cascade is already well established^16^, and based on the current results showing the effect of ficolin-2 on IL-1β and IL-6 expression, the co-localization of ficolin-2 with both cytokines was investigated by immunofluorescence in atherosclerotic plaque specimens from patients with high plasma levels of ficolin-2 (as described in^10^). As shown in Fig. 7, ficolin-2 co-localizes with both IL-1β (upper panel) and IL-6 (lower panel) in the atherosclerotic plaque.

## Discussions

Recent data suggest a role for the lectin pathway initiator, ficolin-2, in the evolution of atherosclerotic plaques. Ficolin-2 was detected in carotid plaques, specifically in the lipid core, in collagen-rich areas of the tunica media, and in regions abundant in cholesterol crystals, where it is thought to attract macrophages^7,10^. High circulating levels of ficolin-2 have been associated with intraplaque recruitment of immune cells, such as macrophages, neutrophils, mast cells, and lymphocytes^10^, all of which are involved in the vulnerable plaque progression. There is evidence that ficolin-2 has a direct effect on macrophages and induces M1 macrophage polarization^11^, and the interaction between MØ and SMC – cells that are often found close to each other in the plaque^4,5,17^—may contribute to plaque vulnerability. However, the direct effects of ficolin-2 on SMC activation or on the interactions between SMC and macrophages have not yet been examined.

In this context, the main objective of this study was to evaluate the intraplaque cellular and molecular mechanisms mediated by ficolin-2 using an in vitro model of co-cultured human SMC and macrophages.

Our results bring to light novel findings which suggest that ficolin-2, independent of lectin complement pathway activation, intervenes in the cross-talk between SMC and MØ, enhancing inflammation through specific mechanisms. These mechanisms include: (i) SMC activation dependent on the MØ, which results in increased MCP-1 gene expression, IL-6 production, and activation of the ERK1/2 and NF-kB pathway; (ii) upregulation of IL-1β, IL-6, and MIP-1β protein expression in macrophages; (iii) the release of inflammatory mediators IL-1β, IL-6, and MIP-1β in the conditioned media, resulting in increased monocyte chemotaxis.

In this study, we explored the direct involvement of ficolin-2 on SMC, and its effects on SMC and macrophage cross-talk, using an in vitro co-culture model. A growing body of research supports the involvement of complement-dependent inflammation in the pathophysiology of cardiovascular disease^18^. We propose a novel mechanism whereby ficolin-2 contributes to the progression of atherosclerosis independent of complement activation by enhancing inflammation in the cross-talk between the smooth muscle cells and macrophages, key cells in the evolution of atherosclerosis.

We found that ficolin-2 does not directly activate SMC in terms of the production of inflammatory mediators, ECM, MMPs, or by altering phenotypic markers. However, when SMC were placed in contact with macrophages and exposed to ficolin-2 in a co-culture setting, there was a noticeable increase in MCP-1 gene expression and IL-6 gene and protein expression, as well as an upregulation of TLR4 protein expression in SMC. These changes were accompanied by the activation of the NF-kB and ERK1/2 signaling pathways in SMC.

Notably, while we observed an increase in MCP-1 gene expression, we did not detect a corresponding increase in MCP-1 protein levels in SMC interacted with macrophages nor a significant release of MCP-1 in the conditioned media. These data may suggest either a different time course for the protein expression or that an additional, stronger stimulus may be required for protein expression. However, we can speculate that in the context of an atherosclerotic plaque, these conditions could be present, thereby ficolin-2 could contribute to the increased MCP-1 expression found in plaque.

Importantly, our data show that ficolin-2 significantly increased the expression of IL-6 in SMC following their interaction with macrophages. IL-6 is known to be expressed in normal arteries as well as in atherosclerotic lesions, and can be increased in SMC in response to various stimuli such as lipopolysaccharide (LPS), interleukin-1 (IL-1), tumor necrosis factor-α (TNFα), endothelin, hypoxia, and aging^19^. Our results extend these data, showing that ficolin-2, a pattern recognition molecule and an initiator of the complement lectin pathway, also amplifies IL-6 production in SMC by a macrophage-mediated mechanism. Ficolin-2 was shown to induce macrophage-mediated effects on immune cells (CD8^+^ T cells)^13^, similar to our findings where ficolin-2 interacts with SMC but does not directly activate them. Considering that dysregulated IL-6 activity is associated with chronic inflammation and atherosclerosis, and that circulating IL-6 is an independent predictor of carotid plaque severity, vulnerability, and progression^20^, our findings suggest that, by increasing IL-6 expression by SMC, ficolin-2 may contribute to the acceleration of plaque progression towards vulnerability. As we also found that ficolin-2 induced a rapid increase and release of IL-1β in macrophages, and there is evidence that IL-1β induces IL-6 in SMC^21^, it is plausible to assume that IL-1β might be partly responsible for the activation of SMC in our model. Furthermore, our data show that ficolin-2 activates NF-kB and ERK1/2, key inflammatory signaling pathways, in SMC following interaction with macrophages. These pathways may be involved in the production of inflammatory mediators by the interacting SMC, although further experiments are needed to clarify this matter.

Intriguingly, ECM proteins and MMP mediators associated with plaque remodeling, a process that is dependent on SMC, were not modified after this relatively short exposure (6 and 24 h) of the co-culture to ficolin-2. However, we should not rule out the possibility that ficolin-2 could impact these processes over longer periods since previously, we found that a longer duration of interaction (24 and 72 h) between SMC-MØ led to a decrease in ECM matrix components synthesis and an increased production of MMPs in SMC^5^.

Regarding macrophages, there is evidence showing that ficolin-2 promotes M1 macrophage polarization and boosts IL-1β production in PMA-stimulated THP-1 cells^11^ and that IL-1β is induced in both MØ and SMC upon their interaction^5^. Our results go further and demonstrate that ficolin-2 amplifies not only IL-1β, but also IL-6, and MIP-1β protein expression in MØ, beyond the levels induced by cell cross-talk alone. These data are in accordance with previous studies showing that MØ and SMC cross-talk considerably augmented MØ cytokine expression^4^ and with recent single-cell RNA-sequencing data revealing that IL-1β serves as a critical modulator of vascular SMC – MØ cross-talk during atherosclerosis progression^22^. In addition, there is supporting evidence that ficolin-2 can bind to MØ in vitro and interact with TLR4, which can trigger MØ activation, promote M1 polarization, and increase IL-1β production^11^.

The cytokine IL-1β is the key initiator of the acute inflammatory response^23^ and contributes to atherosclerosis progression through a variety of complex mechanisms. It can act as a local paracrine and autocrine stimulator, increasing the secretion of multiple cytokines and cell adhesion molecules, which in turn, lead to immune cell extravasation and persistent local inflammation^24^. Additionally, IL-1β can affect vascular SMC and induce the expression of MMPs to accelerate the degradation of the atherosclerotic plaque fibrous collagen skeleton^25,26^.

Therefore, we may propose that ficolin-2, by enhancing IL-1β release during SMC-MØ cross-talk, and potentially within the plaque itself, could intensify both acute and chronic inflammation in the plaque.

In light of the recent CANTOS study, which demonstrated that patients treated with Canakinumab (a monoclonal antibody IL-1β) had a significantly lower incidence of clinical outcomes such as atherosclerosis-related myocardial infarction and stroke, and showed a significant association between the effect of canakinumab and the decreased levels of IL-6^27^, it was suggested a synergistic role of IL-1β and IL-6 in atherosclerosis progression^28^. Given these findings and the evidence that serum levels of ficolin-2 correlate with future major cardiovascular events in patients with carotid atherosclerosis^10^, we might speculate that IL-1β and IL-6 provides a link between ficolin-2 and the occurrence of such events.

The production mechanism of IL-1β can be dependent on TLR4 and there is evidence that the TLR4-activated MAPK-IL-6 axis regulates vascular SMC function^29^. Also, abnormal activation of TLRs is associated with the development and progression of atherosclerosis^30^. Our results show that ficolin-2 increases TLR4 expression in interacting SMC, which raises the possibility that ficolin-2, by enhancing TLR4 expression and activation, may contribute to atheroprogression in the plaque.

As already mentioned, ficolin-2 also has an impact on MIP-1β protein expression in macrophages beyond the levels already induced by cellular cross-talk. As MIP-1β (CCL4) was shown to be elevated in atherosclerotic patients' plasma and plaques^31^ where it correlates with circulating levels of ficolin-2^10^, we suggest that ficolin-2 interference in SMC and MØ cross-talk might contribute to the increased MIP-1β levels found in patients plasma.

The functionality of these increased inflammatory mediators is indicated by enhanced monocyte transmigration towards conditioned media from cells co-cultured in the presence of ficolin-2 as compared with condition media from co-cultured cells only, suggesting that ficolin-2 could intensify the inflammatory process in atherosclerotic plaque by raising monocyte recruitment, thus helping to elucidate the observed correlation in atherosclerotic patients where circulating ficolin-2 levels were found to be correlated with monocyte and macrophages in the plaques^10^. This assumption is sustained by blocking experiments showing that in the presence of ficolin-2 blocking antibody, the number of transmigrated monocytes was reduced (Supplementary Figure S3).

Recent data showing that higher concentrations of PTX3 and ficolin-2 are associated with an increased risk of MI in relatively healthy middle-aged individuals, independent of conventional CVD risk factors, support the involvement of complement-dependent inflammation in the pathophysiology of cardiovascular disease^18^. Similarly, ficolin-2 (and not ficolin-1 or -3) was found to bind to cholesterol crystals in the plaque, triggering the lectin pathway and inflammation, suggesting a contribution of ficolin-2 to atheroprogression^9^. Our results complement these important data and show that ficolin-2 could exacerbate plaque inflammation, independent of complement pathway activation, by modulating the cellular interaction between SMC and MØ in plaque through specific cellular and molecular mechanisms which may lead to plaque destabilization.

Enhanced inflammation has long been associated with plaque vulnerability^32^. SMC and MØ, frequently found to be associated in atherosclerotic plaques, may be significant contributors to plaque inflammation, playing a crucial role in the evolution towards a stable or vulnerable plaque.

### Study limitations

Our study has several limitations that need to be taken into account. First, the absence of in vivo experiments to validate the in vitro findings, and secondly our in vitro model which falls short in fully replicating the intricate complexity inherent to atherosclerosis. In addition, the timeframes of our experiments do not definitively ascertain its suitability for investigating either the acute or long-term aspects of atherosclerosis. Despite these limitations, our findings provide important insights into the inflammatory ficolin-2 mechanisms in smooth muscle cells and macrophages cross-talk, and may explain the data obtained in human atherosclerotic patients studies^10^.

In conclusion, our study indicates that ficolin-2, independent of complement activation, could enhance the inflammatory process within atherosclerotic plaque by modulating the SMC and MØ cross-talk and by increasing monocyte transmigration, SMC inflammation and IL-1β and IL-6 production. These insights open up new perspectives on the cellular and molecular dynamics in atherosclerotic plaque progression and highlight potential therapeutic targets for mitigating cardiovascular disease.

### Supplementary Information

Supplementary Information.

## Data Availability

The datasets used and/or analysed during the current study available from the corresponding author on reasonable request.
