# Supplementary material for: Ficolin-2 amplifies inflammation in macrophage-smooth muscle cell cross-talk and increases monocyte transmigration by mechanisms involving IL-1β and IL-6
Source: Sci Rep. 2023 Nov 8;13:19431. doi: 10.1038/s41598-023-46770-0 (PMC10632380; doi:10.1038/s41598-023-46770-0)

Supplementary data

Table 1. List of genes quantified by RealTime PCR. The table shows the primer pairs used in the amplification reaction.

Gene	Forward primer	Reverse primer
IL-1β	AGAGTGGAGCCTGGTCTTACA	CCTTTGCTGACAATAAGCACTGG
IL-6	CCTGAACCTTCCAAAGATGGC	TTCACCAGGCAAGTCTCCTCA
TNFα	AGCATGAAAGTCTCTGCCGCCCTTCTG	CGGGCCGATTGATCTGAGC
MCP-1	ATTACTTAAGGCATAATGTTTCACA	AGCATGAAAGTCTCTGCCGCCCTTCTG
GM-CSF	TCCTGAACCTGAGTAGAGACAC	TGCTGCTTGTAGTGGCTGG
E-selectin	GGGAAGATGGTCGTGATCCTT	TCTGGGGTGGTCTCGATTTTA
VCAM1	TTGGGCATAGAGACCCCGTT	GCACATTGCTCAGTTCATACACC
MIP1β	CTGTGCTGATCCCAGTGAATC	TCAGTTCAGTTCCAGGTCATACA
CCL5	CCAGCAGTCGTCTTTGTCAC	CTCTGGGTTGGCACACACTT
IL18	GATAGCCAGCCTAGAGGTATGG	CCTTGATGTTATCAGGAGGATTCA
TLR4	ACCTGAGCTTTAATCCCCTGA	GGCTCTGATATGCCCCATCTT
TLR2	CGGGCCGATTGATCTGAGC	GGACAGGTCAAGGCTTTTTACA
MMP8	AAGTGGGAACGCACTAACTTG	GGATTCCATTGGGTCCATCAAAT
MMP13	ACTGAGAGGCTCCGAGAAATG	GAACCCCGCATCTTGGCTT
MMP9	AGACCTGGGCAGATTCCAAAC	CGGCAAGTCTTCCGAGTAGT
LOX (Lysyl oxidase)	CACTATGACCTGCTTGATGCCA	GATGTCCTGTGTAGCGAATGTC
THBS1 (Thrombospondin1)	AGACTCCGCATCGCAAAGG	TCACCACGTTGTTGTCAAGGG
Collagen I	AATTGGAGCTGTTGGTAACGC	CACCAGTAAGGCCGTTTGC
Collagen IV	GGACTACCTGGAACAAAAGGG	GCCAAGTATCTCACCTGGATCA
Collagen III	TTGAAGGAGGATGTTCCCATCT	ACAGACACATATTTGGCATGGTT

OPN (Osteopontin)	GAAGTTTCGCAGACCTGACAT	GTATGCACCATTCAACTCCTCG
αSMA	ACTGCCTTGGTGTGTGACAA	CACCATCACCCCCTGATGTC
CD68	GAAACGTCACAGTTCATCCAACA	ACGTGTAGTCTCCAATGGTCTC

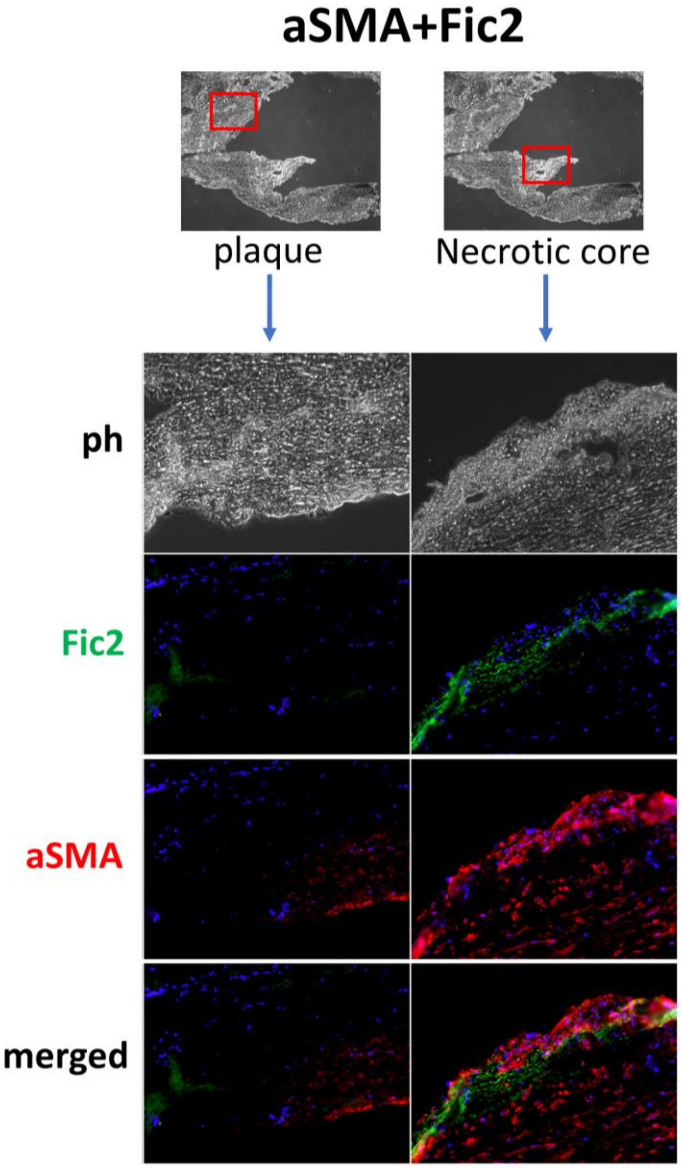

Supplementary Figure 1. Immunofluorescence images staining ficolin-2 and SMC marker αSMA on sections obtained from some region (left column) or from the necrotic core region (right column) of the atherosclerotic plaque.

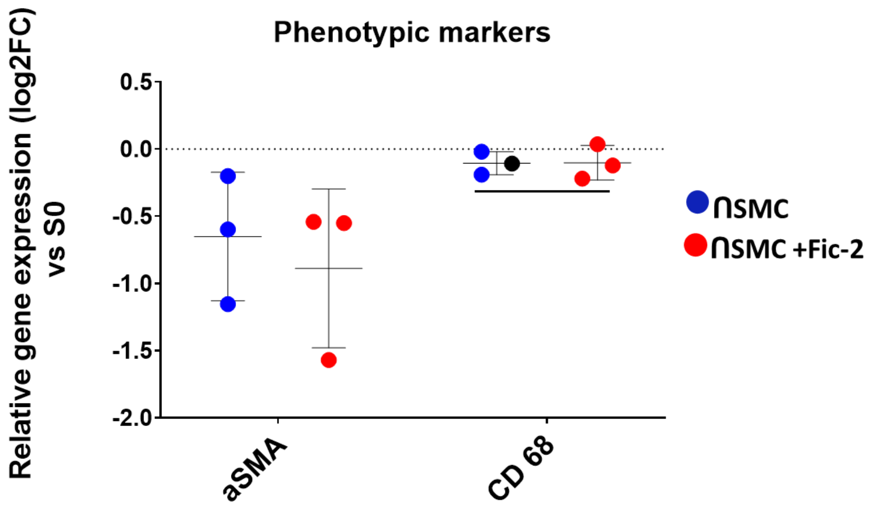

Supplementary Figure 2. Relative gene expression (log2FC vs SMC) of phenotypic markers αSMA and CD68 in interacted SMC in the presence or absence of ficolin-2, compared with control SMC (dotted line) (n=3).

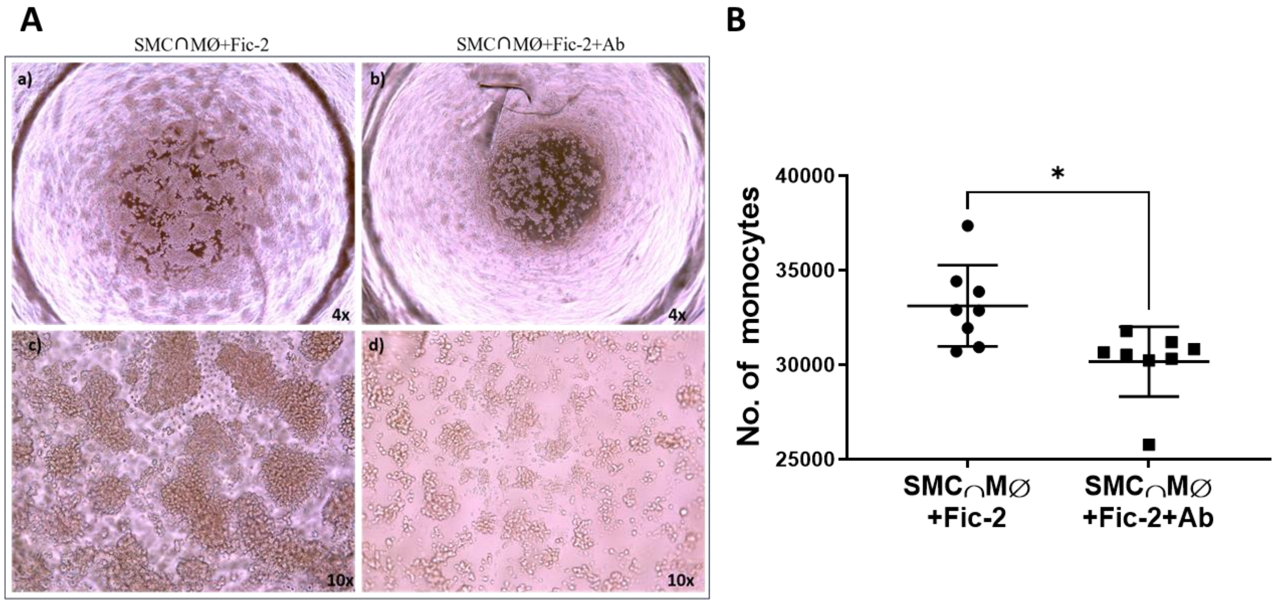

Supplementary Figure 3. (A) Monocytes transmigration towards conditioned media from SMCႶMØ+Fic-2 (a and c) or towards the same conditioned media treated with antibody to Ficolin-2 (b and d). Representative optical microscopy images of monocytes that transmigrated in the lower chamber of the trans-well plate at two magnifications, 4x (a and b) and 10x (c and d). **(B)** Quantification of the monocytes number transmigrated towards SMCႶMØ+Fic-2 or SMCႶMØ+Fic-2+Ab; $p<0.05$, n=8.

Supplementary data
Original blots

Figure 2C

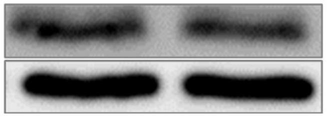

For Figure 2C, a replicate of the experimental data shown in the manuscript is provided.

1

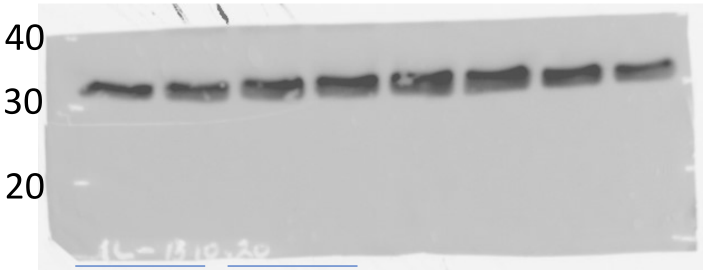

2

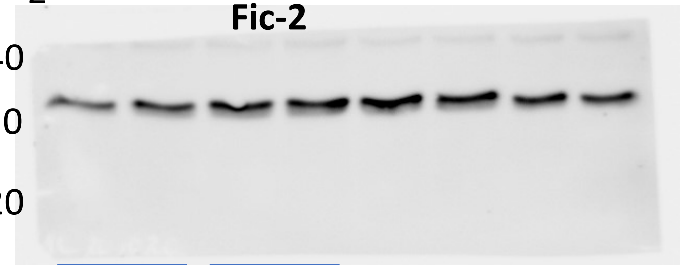

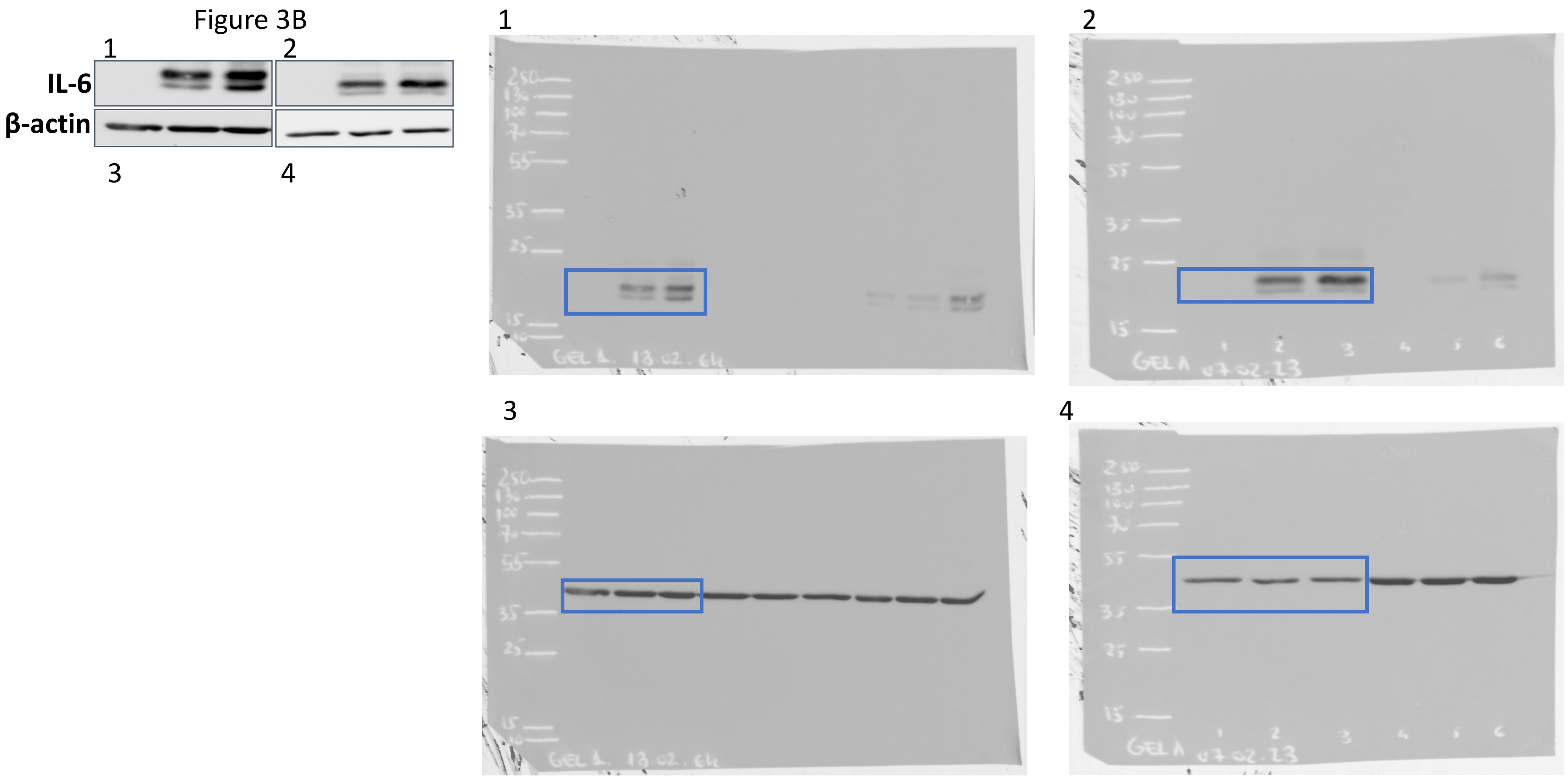
Figure 3B
IL-6
β-actin
1
2
3
4

Figure 3C

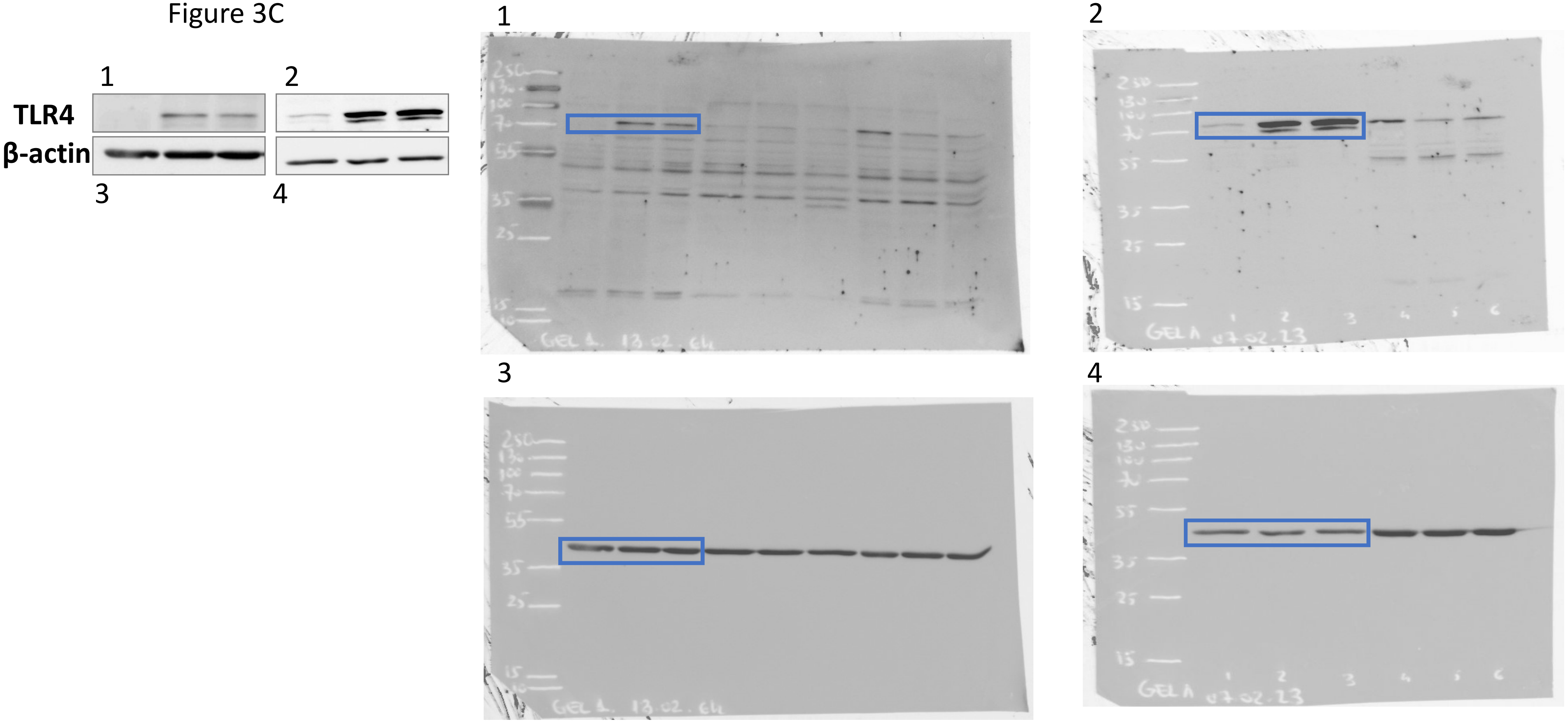

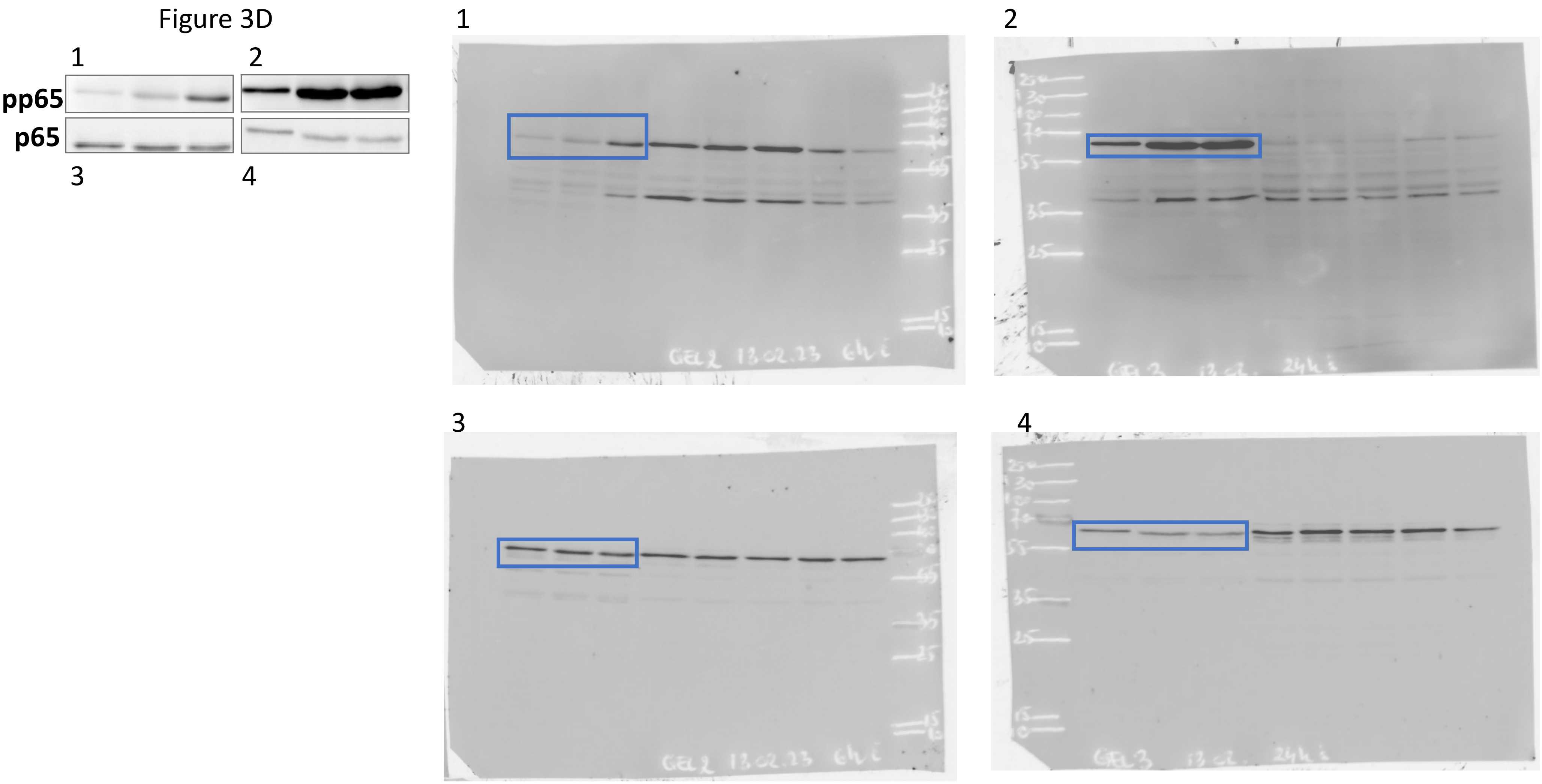
Figure 3D
1
2
pp65
p65
3
4
1
2
3
4

Figure 3E

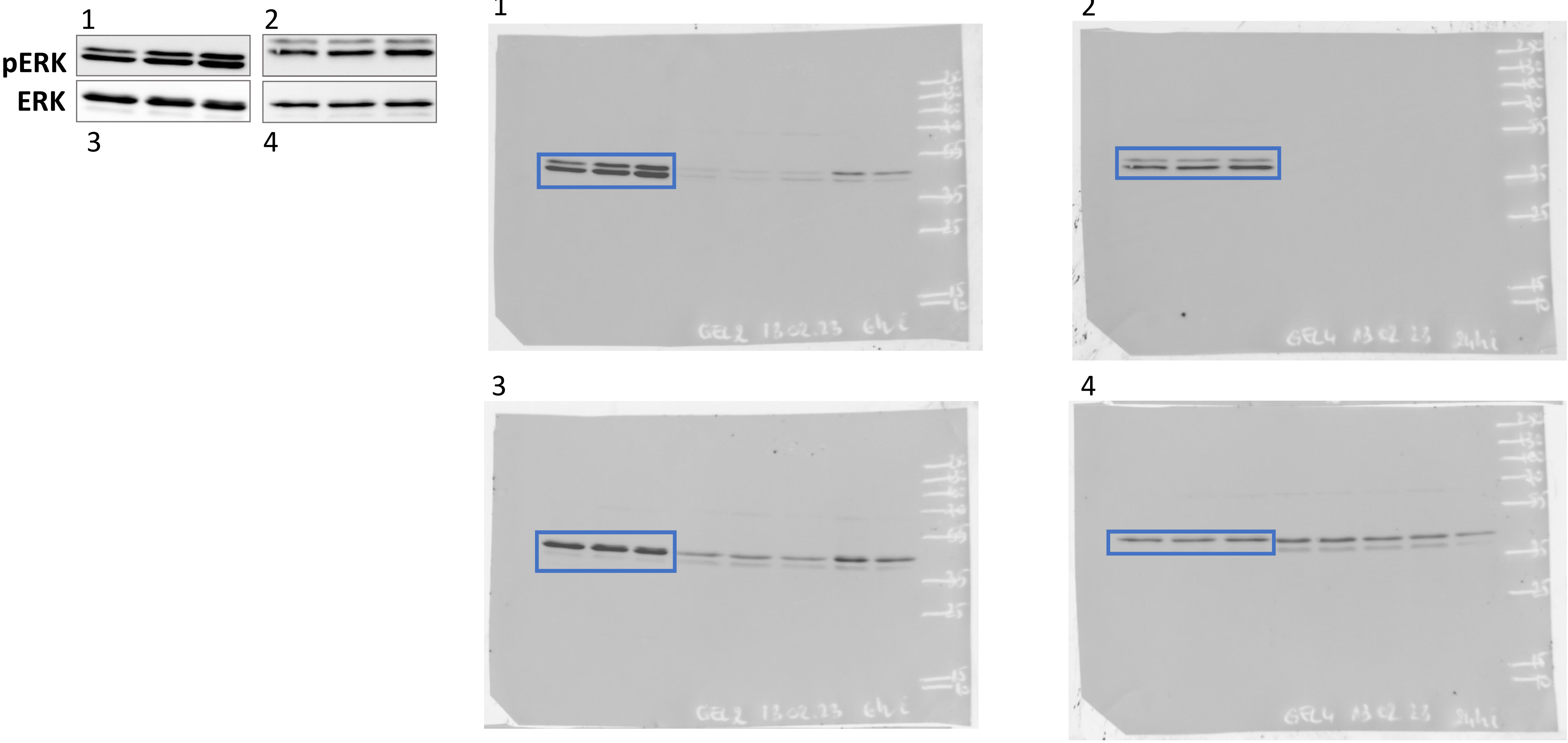

Figure 3F

1 2

pp38
p38

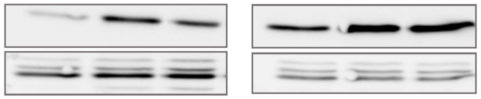

3 4

1

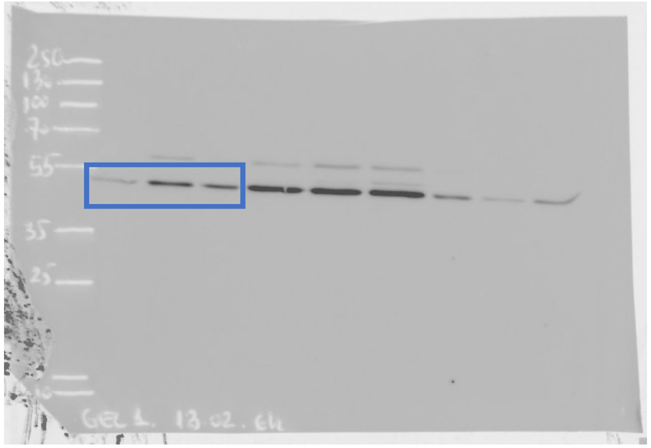

2

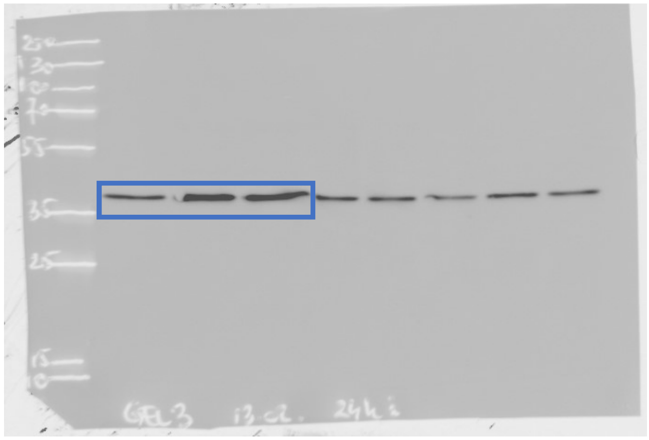

3

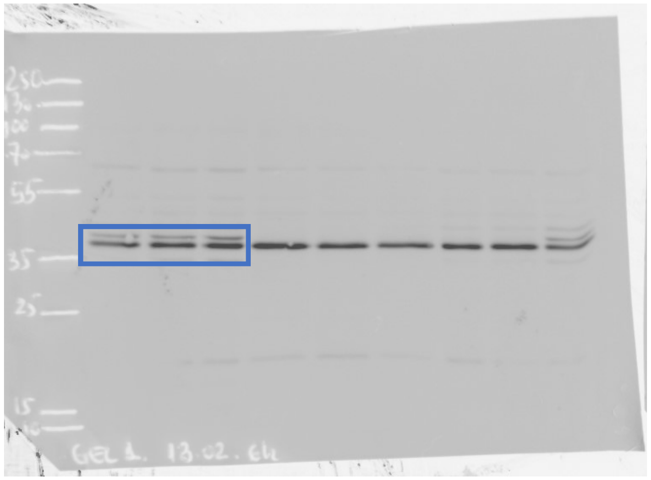

4

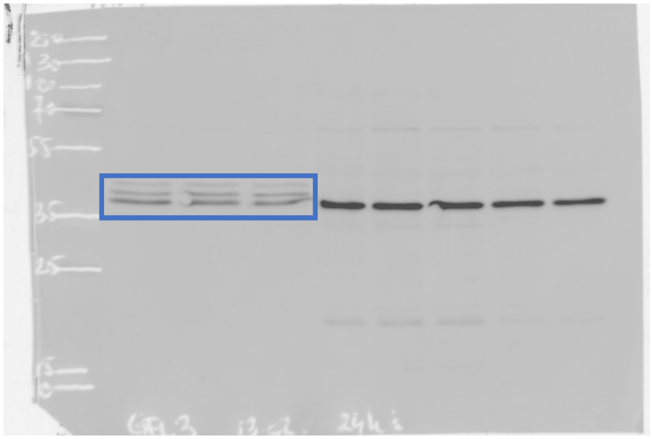

Figure 3G

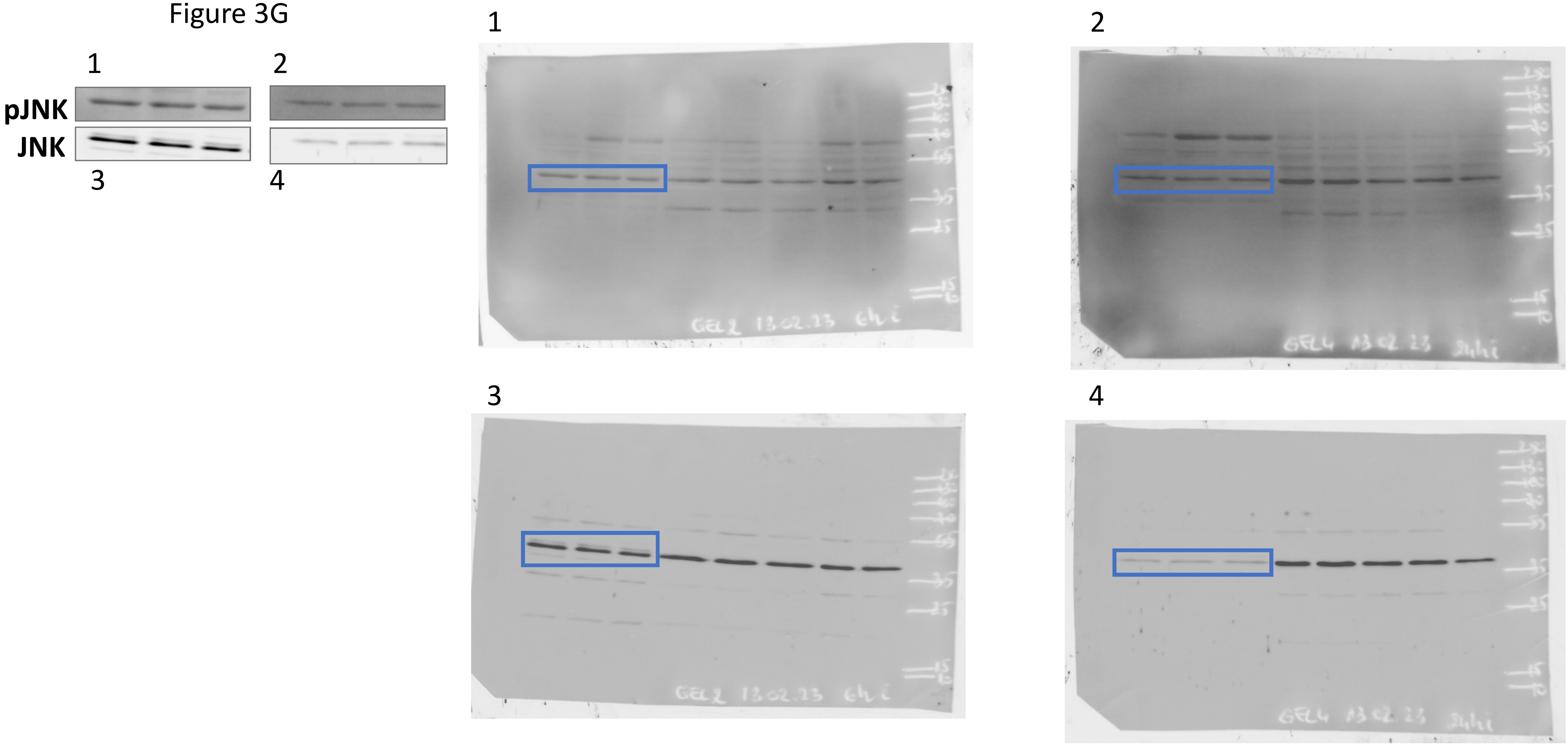

Figure 5A

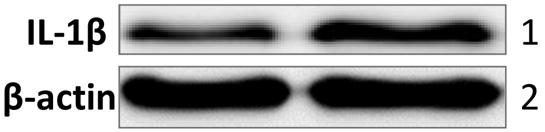

1

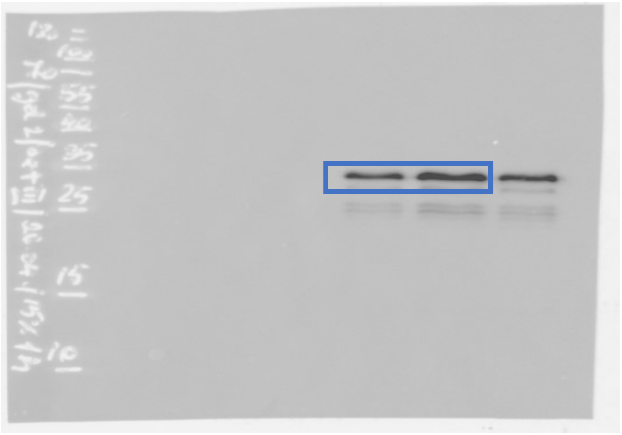

2

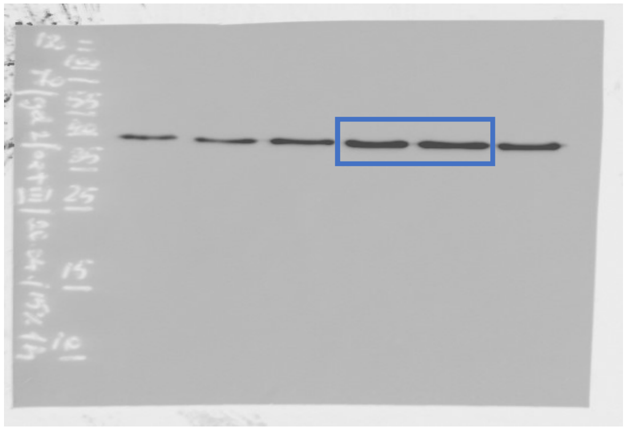

Figure 5B

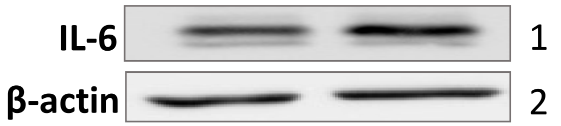
IL-6
β-actin
1
2

1

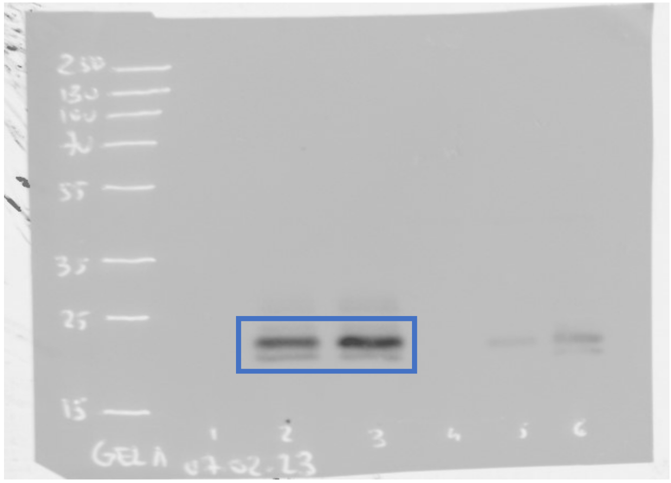
250
130
100
70
55
35
25
15
1
2
3
4
5
6
GEL A 07.02.23

2

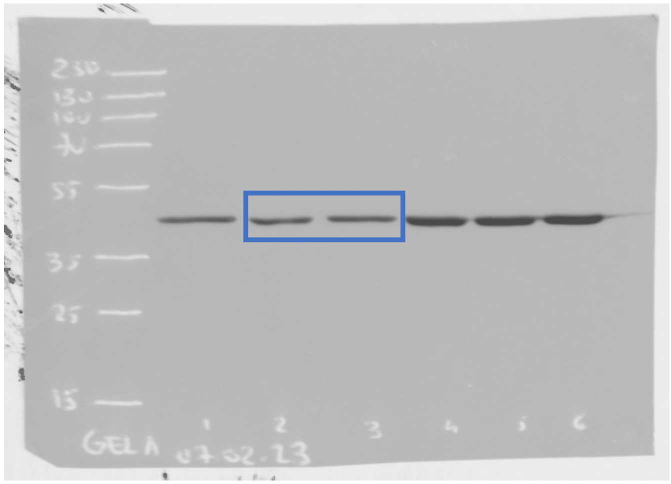
250
130
100
70
55
35
25
15
1
2
3
4
5
6
GEL A 07.02.23

Figure 5C

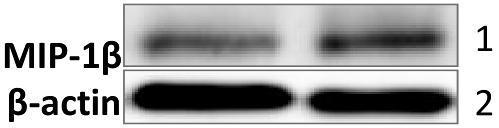

1

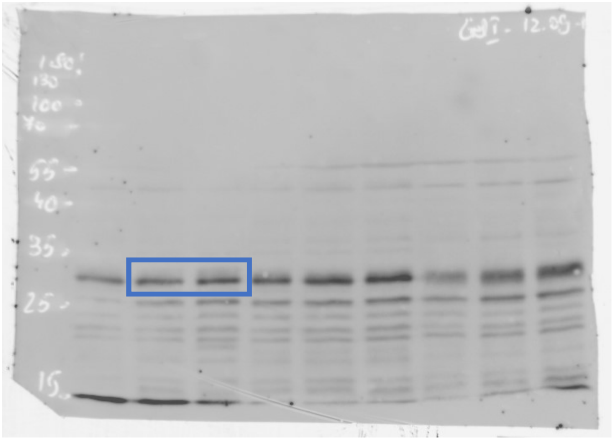

2

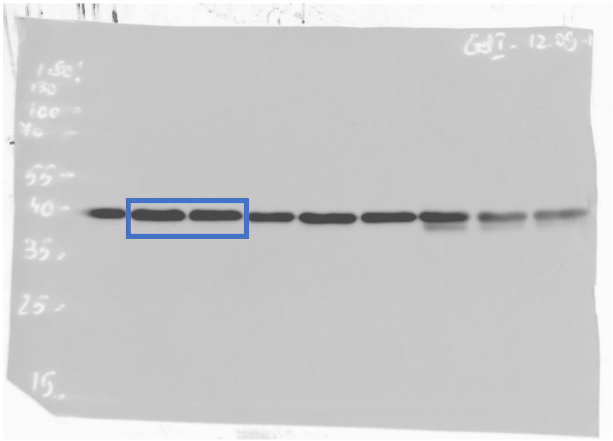

Supplement: Supplementary file 1 — Supplementary Information. [file 41598_2023_46770_MOESM1_ESM.pdf]
